# Supplementary material for: Rapid turnover of life-cycle-related genes in the brown algae
Source: Genome Biol. 2019 Feb 14;20:35. doi: 10.1186/s13059-019-1630-6 (PMC6374913; doi:10.1186/s13059-019-1630-6)
Supplement: Supplementary file 3 — Protein alignments, corresponding Gblocks html file and CDS sequences for the lineage conserved gametophyte-biased genes. (ZIP 2109 kb) [file 13059_2019_1630_MOESM3_ESM.zip › Con_GA_alignments/Ec-07_003830.aa_ali.fasta-gb.htm]

Ec-07\_003830.aa.fasta.trans.aa\_ali.fasta


## Gblocks 0.91b Results

Processed file: **Ec-07\_003830.aa.fasta.trans.aa\_ali.fasta**  
Number of sequences: **2**  
Alignment assumed to be: **Protein**  
New number of positions: **334** (selected positions are underlined in blue)

```
                         10        20        30        40        50        60
                 =========+=========+=========+=========+=========+=========+
Ec-07_003830     MRKYRLVAKKGEGTFSEVLKAQNVKDGKYYAIKCMKNRFDSIDQVNNLREIQALRRLSPN
MSTRG.37397.1    -RKYRLVAKKGEGTFSEVLKAQNVKDGKYYAIKCMKNRFDSIDQVNNLREIQALRRLSPN
                  ###########################################################


                         70        80        90       100       110       120
                 =========+=========+=========+=========+=========+=========+
Ec-07_003830     QHVITLEEVLYDQPTGRLALVFELMDANLYELIRGRRHYLNGQLVRSYMYQLLKALDHMH
MSTRG.37397.1    QHVITLEEVLYDQPTGRLALVFELMDANLYELIRGRRHYLNGQLVRSYMYQLLKALDHMH
                 ############################################################


                        130       140       150       160       170       180
                 =========+=========+=========+=========+=========+=========+
Ec-07_003830     RKGIFHRDIKPENILIESTNDLGRGLKLADFGSCRGIYSKQPYTEYISTRWYRAPECLLT
MSTRG.37397.1    RKGIFHRDIKPENILIESTNDLGRGLKLADFGSCRGIYSKQPYTEYISTRWYRAPECLLT
                 ############################################################


                        190       200       210       220       230       240
                 =========+=========+=========+=========+=========+=========+
Ec-07_003830     DGYYGPEMDQWGAGCVFFEITSLYPLFPGTNELDQIGRVHKVLGNPSGEVLGKFKQNGAA
MSTRG.37397.1    DGYYGPEMDQWGAGCVFFEITSLYPLFPGTNELDQIGRVHKVLGNPSGEVLGKFKRNGAA
                 ############################################################


                        250       260       270       280       290       300
                 =========+=========+=========+=========+=========+=========+
Ec-07_003830     HVDFDFATQKGIGVAQLIPHASTECVDLITKLLRYDWSDRCTARESLRHPYFREIREVDL
MSTRG.37397.1    HVDFDFATQKGIGVAQLIPHAATECVDLITKLLRYDWSDRCTARESLRHPYFREIREVDL
                 ############################################################


                        310       320       330       340       350       360
                 =========+=========+=========+=========+=========+=========+
Ec-07_003830     RKAQAESTSKLARGTTEDAAGGGDPATVASTGRAPQTRGAPGGGGGGGGGANHGTGAGGG
MSTRG.37397.1    RKAQAESSSKLARGSTEEAGGG-DADTVASTGRAPQLAHTSSLADAGQSNKALPT-----
                 ###################### #############                        


                        370       380       390       400       410       420
                 =========+=========+=========+=========+=========+=========+
Ec-07_003830     AGGGGGGGGRYRGQAVGQGGSKASSKKKKKNRGYYDSNIRSKYNGMSNGGGGGGGGGGGN
MSTRG.37397.1    ---------------IGQQ------------------------------GGAHGGGGNA-
                                                                             


                        430       440       450       460       470       480
                 =========+=========+=========+=========+=========+=========+
Ec-07_003830     GFGGAKGHRITSRSSFSLMQAAGGGGGAMGSNIGYGGNGGAGGGNGVPGGMAAQAPERAR
MSTRG.37397.1    ------------------------------------------------------------
                                                                             


                        490
                 =========+==
Ec-07_003830     VGKNTSRYRQLR
MSTRG.37397.1    ---NTSG----A
```

```
Parameters used
Minimum Number Of Sequences For A Conserved Position: 2
Minimum Number Of Sequences For A Flanking Position: 2
Maximum Number Of Contiguous Nonconserved Positions: 8
Minimum Length Of A Block: 10
Allowed Gap Positions: None
Use Similarity Matrices: Yes
```

```
Flank positions of the 2 selected block(s)
Flanks: [2  322]  [324  336]  

New number of positions in Ec-07_003830.aa.fasta.trans.aa_ali.fasta-gb:  334  (67% of the original 492 positions)
```
